# A method for functional testing constitutive and ligand-induced interactions of lysin motif receptor proteins

**DOI:** 10.1186/s13007-020-0551-4

**Published:** 2020-01-16

**Authors:** Chun-Lian Li, De-Xing Xue, Yi-Han Wang, Zhi-Ping Xie, Christian Staehelin

**Affiliations:** State Key Laboratory of Biocontrol and Guangdong Key Laboratory of Plant Resources, School of Life Sciences, Sun Yat-Sen University, East Campus, Guangzhou, 510006 Guangdong China

**Keywords:** Chimeric receptor, *Lotus japonicus*, Lysin motif (LysM), LysM receptor-like kinase (LYK), Receptor function

## Abstract

**Background:**

Plant receptors with lysin motifs (LsyM) recognize microbial signals such as fungal chitin and lipo-chitooligosaccharidic Nod factors of nitrogen-fixing rhizobia. It is generally assumed that ligand-induced dimerization of LysM receptors is an essential step in activation of intracellular kinase domains and downstream signaling. Consequently, genes required for plant defense and establishment of symbiosis are expressed. We recently found that three LysM receptor proteins (namely LYK1, LYK4 and LYK5) of *Arabidopsis thaliana* form a tripartite receptor complex to perceive chitin. However, constitutive and ligand-induced interactions of LysM receptors generally remain difficult to be characterized.

**Results:**

Interactions between ectodomains of LYK1, LYK4 and LYK5 were investigated by a chimeric receptor approach using hairy roots of the legume *Lotus japonicus*. Synthetic receptor pairs consisting of a LYK ectodomain and the intracellular domain of a *L. japonicus* Nod factor receptor (NFR1 and NFR5, respectively) were tested for their capacity to activate expression of the symbiotic *NIN* (nodule inception) gene. The results indicated constitutive (LYK4^ED^–LYK4^ED^, LYK4^ED^–LYK5^ED^) and chitin-induced interactions (LYK1^ED^–LYK1^ED^, LYK1^ED^–LYK5^ED^) of the examined ectodomains.

**Conclusion:**

We present a method to functionally analyze constitutive and ligand-induced interactions of LysM-type proteins.

## Background

Plants possess specific receptors and signal cascades to alter gene expression in response to invading microbes. PAMPs (pathogen-associated molecular patterns) are conserved elicitor molecules such as fungal chitin and chitooligosaccharides, bacterial peptidoglycan, and flagellin [1–3]. PAMPs are typically perceived by corresponding PRRs (pattern recognition receptors) in plant plasma membranes. Many PRRs are receptor kinases consisting of an extracellular, ligand-binding ectodomain, a transmembrane domain, and an intracellular kinase domain. Some PRRs lack an intracellular kinase domain or possess an inactive kinase domain. This implies that these receptors require cooperation with another PRR or an adaptor kinase. PRR-mediated signaling results in activation of PAMP-triggered defense reactions (plant immunity) [1, 2, 4]. To recognize symbiotic microbes, host plants perceive specific microbial signals to activate expression of symbiosis-related plant genes. Nitrogen-fixing bacteria, commonly referred to as rhizobia, induce nodules on roots of legumes while nutrient-acquiring (mainly phosphorus) mycorrhizal fungi establish a root symbiosis with most land plants. Rhizobial lipo-chitooligosaccharides, commonly known as Nod factors, and chitinous mycorrhizal molecules (chitin, chitooligosaccharides and lipo-chitooligosaccharides) are key signals to induce plant responses required for nodulation and mycorrhization [5, 6].

Numerous PRRs of plants possess LysM domains in their ectodomains. The LysM domain (Pfam PF01476) has been originally discovered in lysins of bacteriophages and bacteria [7]. LysM receptors of plants are implicated in perception of microbial signals containing *N*-acetylglucosamine such as fungal chitin (or chitooligosaccharides), Nod factors and bacterial peptidoglycan [8–10]. Moreover, specific LysM-type proteins may function as receptors for the oligosaccharidic repeating subunit of rhizobial exopolysaccharide [11] or fungal *β*-1,3-glucans [12]. The Nod factor receptors NFR1 and NFR5 of the model legume *Lotus japonicus* represent the first characterized plant LysM receptors [13, 14]. NFR1 and NFR5 bind Nod factors with high affinity (*K*_d_ values in the nanomolar range) [15]. The protein kinase activity of NFR1 (NFR5 lacks kinase activity) was found to be required for receptor autophosphorylation and subsequent activation of Nod factor signaling [16]. Ligand-induced activation of the Nod factor receptor pair mediates induction of Nod factor signaling that includes various downstream components such as symbiosis receptor kinase (SymRK), calcium and calmodulin dependent protein kinase (CCaMK), its phosphorylation target (named CYCLOPS) and the key transcription regulator (nodule inception protein) NIN required for expression of Nod factor-induced genes [5, 17]. *NIN* expression is strongly up-regulated by Nod factor signaling in *L. japonicus* roots [17, 18].

Specific LysM-type proteins function as receptors or co-receptors for fungal chitin and chitooligosaccharides [4, 9, 10]. The LysM domain-containing protein OsCEBiP (CHITIN ELICITOR-BINDING PROTEIN) of rice (*Oryza sativa*) was the first discovered chitin receptor [19]. CEBiP is a PRR with a glycosylphosphatidylinositol tail anchored to the plasma membrane. When rice cells are elicited by chitin or chitooligosaccharides, CEBiP forms a heterodimeric complex with CERK1 (CHITIN ELICITOR RECEPTOR KINASE 1; a LysM domain-containing PRR with a kinase domain) [20–23]. Similar chitin receptors have been also identified in other plants including legumes (e.g., LjLYS6 in *L. japonicus* [24]). In *Arabidopsis thaliana*, three LysM-type PRR proteins, namely LYK1, LYK4 and LYK5, are implicated in perception of chitin and long-chain chitooligosaccharides (6-8 *N*-acetylglucosamine residues) to activate plant defense reactions. The LYK1 protein (also named AtCERK1) possesses an active kinase domain which is indispensable for chitin signaling. Analysis of *lyk1* mutants showed that LYK1 plays a crucial role in immunity against pathogenic fungi [25–35]. LYK4 is a chitin-binding protein with an inactive kinase domain. Work on *lyk4* mutants indicated that LYK4 partially contributes to chitin-triggered immunity [31, 32, 35]. LYK5 has been identified as a receptor with a very high affinity to chitooligosaccharides. The kinase domain of LYK5 appears to lack protein kinase activity [32]. LYK5 can interact with LYK1 and chitin-induced heterodimerization of these proteins seems to trigger activation of the LYK1 kinase domain. Compared to wild-type plants, *lyk5* mutant plants were partially insensitive to chitin and also showed increased susceptibility to fungal pathogens [32, 35]. Immunoprecipitation assays and bimolecular fluorescence complementation (BiFC) analysis of protoplasts indicated constitutive LYK4–LYK4, LYK5–LYK5 and heterodimeric LYK4–LYK5 interactions. When the protoplasts were elicited with chitin or chitoheptaose (hepta-*N*-acetylchitoheptaose), a tripartite LYK1–LYK5–LYK4 chitin receptor complex could be immunoprecipitated. These findings suggested that, upon ligand binding, LYK5 of LYK4–LYK5 (or LYK5–LYK5) interacted with LYK1 to induce activation of the LYK1 kinase domain. However, constitutive and ligand-induced interactions of LysM receptor protein pairs generally remain difficult to identify.

Here, we present a method for analysis of interactions between LysM-type receptor proteins. We used the ectodomains of LYK1, LYK4, LYK5 as examples to functionally analyze constitutive and ligand-induced receptor interactions *in planta*. The method is based on expression of chimeric receptors in *L. japonicus* roots and expression analysis of the symbiotic marker gene *NIN* [33]. In this system, each chimeric receptor construct consist of an ectodomain from a given LysM-type protein and the intracellular domain from a *L. japonicus* Nod factor receptor protein (NFR1^ID^ and NFR5^ID^, respectively). Constitutive or ligand-induced dimerization of functional receptor pairs results in activation of *NIN* expression as estimated by a co-expressed *NIN* promoter*-β-*glucuronidase fusion (*NIN*p*-GUS* construct).

## Methods

### Plant material

*Lotus japonicus* (ecotype Miyakojima MG-20) and the Nod factor receptor mutants *nfr1-1* and *nfr5-2* (ecotype Gifu) were used for expression of chimeric receptors. The *nfr1-1* and *nfr5-2* mutants (deficient in functional NFR1 and NFR5 proteins) were kindly provided by Simona Radutoiu and Jens Stougaard (Aarhus University, Aarhus, Denmark) [13, 14]. The *nfr1-1* possesses a stop codon in the kinase domain VIII [13] and the *nfr5*-*2* mutant contains a retrotransposon insertion generating a truncated protein lacking the C-terminal kinase domain [14]. Seeds were treated with concentrated sulfuric acid (10 min), washed with sterile distilled water, 70% (v/v) ethanol (1 min) and then with sterile water again. The seeds were surface-sterilized with sodium hypochlorite (0.05% active chlorine; 5 min), extensively washed with sterile water and then used for hairy root transformation.

### DNA constructs

**Fig. 1:**
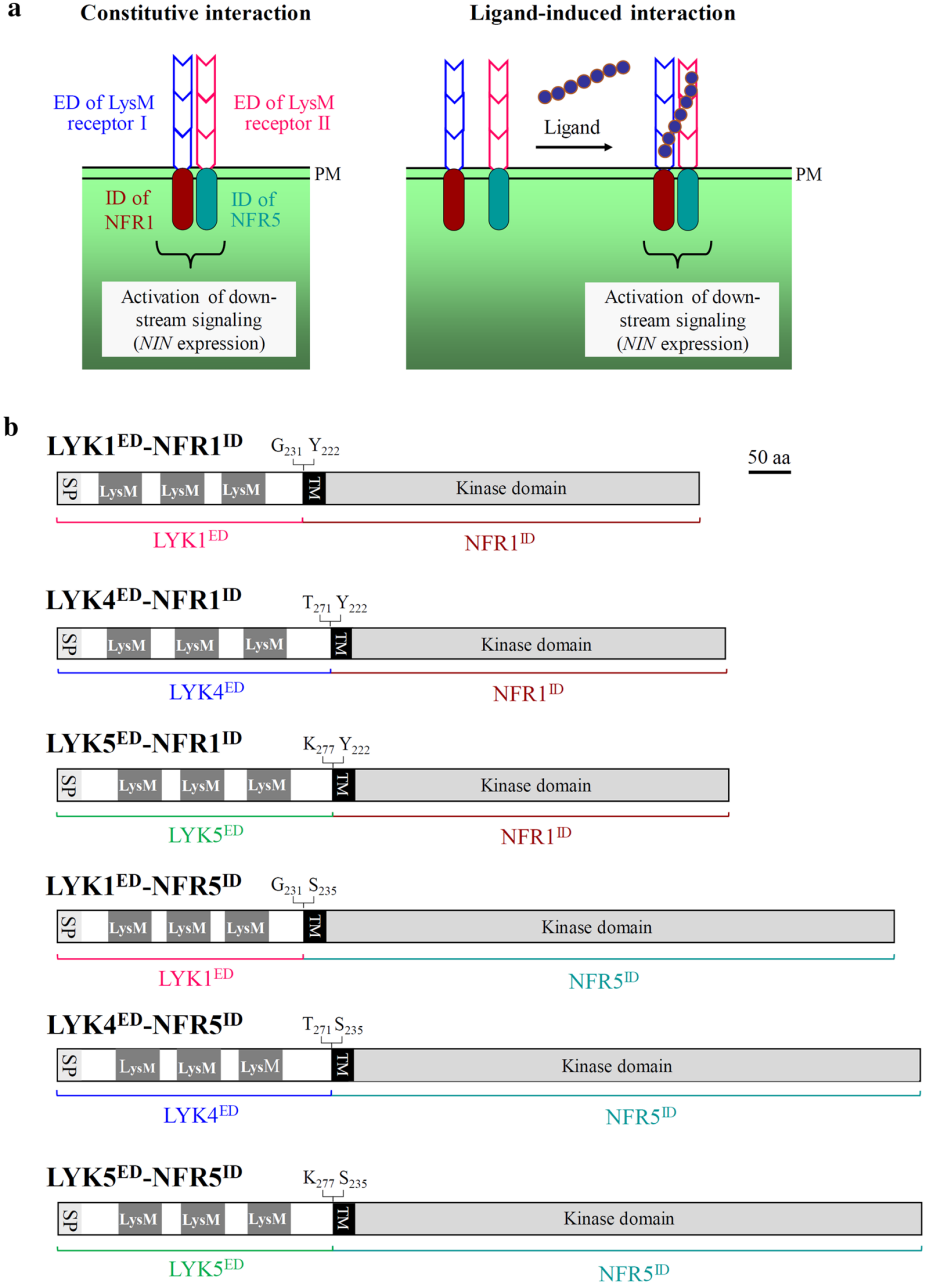
Schematic representation of chimeric receptors expressed in *L. japonicus*. **a** Each hybrid receptor contains an ectodomain of a given LysM receptor and an intracellular domain of a *L. japonicus* Nod factor receptor protein (NFR1 and NFR5, respectively). Constitutive or ligand-induced interactions of ectodomains result in downstream signaling and *NIN* expression. **b** Chimeric receptors used in this study. Letters followed by numbers indicate positions of amino acids forming the fusion points. The kinase domain of NFR1^ID^ is functional and that of NFR5^ID^ is likely non-functional. *PM* plasma membrane, *SP* signal peptide, *LysM* LysM domain, *TM* transmembrane region, *ED* ectodomain, *ID* intracellular domain

To estimate *NIN* expression in *L. japonicus* roots, a 2722-bp *NIN* promoter sequence from *Lotus japonicus* MG-20 was inserted into the pCAMBIA1305.1 vector (https://www.cambia.org) lacking the original CaMV 35S promoter sequence. The *β-*glucuronidase (GUS) gene in this vector (also known as *GUSPlus*) contains an intron of a catalase gene from castor bean to prevent expression by *A. rhizogenes* and ensure detection of GUS activity expressed by *L. japonicus*. The resulting plasmid (pCAMBIA-*NIN*p) containing a *NIN* promoter*-β-*glucuronidase fusion (*NIN*p*-GUS*) was then used as an acceptor for chimeric receptor constructs. The hybrid protein constructs consisting of an *A. thaliana* LYK ectodomain (LYK1^ED^, 693-bp fragment of *LYK1*; LYK4^ED^, 813-bp fragment of *LYK4*; LYK5^ED^, 831-bp fragment of *LYK5*) and an intracellular domain of a *L. japonicus* Nod factor receptor of (NFR1^ID^, 1203-bp fragment of *NFR1*; NFR5^ID^, 1086-bp fragment of *NFR5*) were obtained by overlap extension PCR. The constructs (namely LYK1^ED^–NFR1^ID^, LYK4^ED^–NFR1^ID^, LYK5^ED^–NFR1^ID^, LYK1^ED^–NFR5^ID^, LYK4^ED^–NFR5^ID^, LYK5^ED^–NFR5^ID^) were inserted into pRT104 [36] to yield expression cassettes containing a CaMV 35S promoter and a poly-A tail. The chimeric receptor pair constructs were then introduced into the pCAMBIA-*NIN*p vector by using the Hieff Clone™ Plus Multi One Step Cloning Kit (Yeasen, Shanghai, China). The resulting binary vectors contained expression cassettes encoding the following chimeric receptor pairs: LYK1^ED^–NFR1^ID^/LYK1^ED^–NFR5^ID^, LYK1^ED^–NFR1^ID^/LYK4^ED^–NFR5^ID^, LYK1^ED^–NFR1^ID^/LYK5^ED^–NFR5^ID^, LYK4^ED^–NFR1^ID^/LYK4^ED^–NFR5^ID^ and LYK5^ED^–NFR1^ID^/LYK4^ED^–NFR5^ID^. Moreover, pCAMBIA-*NIN*p with the expression cassette encoding LYK4^ED^–NFR5^ID^ was constructed. Finally, all generated binary vectors were used for hairy root transformation of *L. japonicus.* Primers and plasmids used are listed in Additional file 1: Tables S1 and S2. Schematic views of expressed chimeric proteins are shown in Fig. 1.

### Hairy root transformation

The pCAMBIA-*NIN*p derivatives containing chimeric receptor constructs were mobilized into *Agrobacterium rhizogenes* strain LBA9402 [37] by electroporation. Bacteria were grown in YMB medium (2 g/L mannitol, 0.4 g/L yeast extract, 0.1 g/L NaCl, 0.66 g/L K_2_HPO_4_·3H_2_O, 0.2 g/L MgSO_4_·7H_2_O and 15 g/L agar; pH 7) supplemented with corresponding antibiotics. Transgenic hairy roots of *L. japonicus* plants (*L. japonicus* MG-20, Nod factor receptor gene mutants *nfr1-1* and *nfr5-2*) were obtained according to procedures described previously [33, 38]. Briefly, germinated seedlings (with a root length of about 1 cm) were placed on 0.8% (w/v) agar (HKM, Guangzhou, China) plates containing Gamborg’s 1/2 B_5_ Salts and Vitamins medium (Sigma-Aldrich). The plates were kept in a temperature-controlled growth room (~ 24 °C; 16-h photoperiod; ~ 2000 lx light intensity, Philips Lifemax TL-D 36W/54-765 and TL-D 36W/29-530 daylight fluorescent tubes at a ratio 3:1). The plates were partially covered with aluminum foil to protect the roots from light. After incubation for 24 h, roots of seedlings were diagonally cut off by a sterile scalpel and the hypocotyls of the wounded seedlings were immersed in a given *A. rhizogenes* suspension at 24 °C for 30 min. The transformed seedlings were then transferred to new agar plates. The plates were placed at an angle of approximately 75° and kept in the temperature-controlled growth room. Seedlings were transferred to freshly prepared plates every week. Three weeks after transformation, plants with formed hairy roots were used for elicitor treatments, GUS staining and gene expression analysis.

### Elicitor treatment and GUS staining

Transformed *L. japonicus* roots were treated with a suspension containing 1 μM chitoheptaose (hepta-*N*-acetylchitoheptaose; Elicityl, Crolles, France) or 10 μg/mL chitin (Sigma-Aldrich) for 4 h. Control plants were treated with corresponding amounts of sterile water. Finally, harvested roots were subjected to histochemical GUS staining using 5-bromo-4-chloro-3-indol-glucuronide cyclohexylamine salt [39]. Briefly, *L. japonicus* roots were transferred to 10-mL tubes, then soaked with GUS staining solution and exposed to a vacuum for 20 min. Samples were incubated at 37 °C for 10 h. Finally, roots were rinsed three times with 10% (v/v) commercial bleach and then washed with sterile water. Roots were observed by a stereo microscope and photographed (Lumar.V12, Zeiss, Oberkochen, Germany). Hairy roots with clearly visible blue coloration in at least some root regions were considered as GUS-positive. Transformation efficiency was estimated based on the ratio of GUS-positive hairy roots to the total number of formed hairy roots.

### Reverse transcription PCR

Expression of chimeric receptor genes in hairy roots of *L. japonicus* was confirmed by reverse transcription PCR. Non-transformed roots were used as a negative control. Root RNA was isolated using a HiPure Plant RNA Mini extraction kit (Magen, Guangzhou, China) following the manufacturer’s instructions. The first-strand cDNA was synthesized using the HiScript II Q RT SuperMix for qPCR Kit (Vazyme, Nanjing, China) according to the provider’s instructions. Reverse transcription PCR was performed with obtained cDNA and primers listed in Additional file 1: Table S1. Ubiquitin (Lj5g3v2060710.1) gene primers were used for control reactions. Following thermocycling conditions were used: (i) 95 °C denaturing for 3 min; (ii) 35 cycles: 95 °C for 30 s, 56 °C for 30 s, 72 °C for 30 s; (iii) 72 °C for 5 min; (iv) 25 °C cooling for 5 min. The PCR products were analyzed by agarose gel electrophoresis. The results are shown in Additional file 2: Figure S1.

### qRT-PCR analysis

Expression of chimeric receptor genes in *L. japonicus* was also analyzed by real-time quantitative reverse transcription PCR (qRT-PCR). Non-transformed roots were used as a negative control. The RNA extractions were performed in triplicate (three independent biological replicates). Isolation of root RNA and synthesis of the first-strand cDNA were conducted as mentioned above. qRT-PCR analysis was performed with obtained cDNAs and primers are listed in Additional file 1: Table S1, primers 32–38. Each cDNA sample was three times PCR-analyzed (three technical replicates). Ubiquitin (Lj5g3v2060710.1) gene primers were used for normalization. qRT-PCR reactions were performed with the ChamQTM SYBR® qPCR Master Mix (Roche, Basel, Switzerland) in a LightCycler® 480 apparatus. Each PCR reaction consisted of 1 μL of cDNA template (50 ng), 0.25 μM of each primer and 5 μL of the SYBR Green I Master Mix in a final volume of 10 µL. Following thermocycling conditions were used: (i) denaturing: 95 °C for 5 min; (ii) 45 cycles: 95 °C for 30 s, 60 °C for 30 s, 72 °C for 10 s; (iii) melting curves: 95 °C for 5 s, 60 °C for 1 min; (iv) 40 °C for 30 s. Threshold cycles (Ct values) were calculated with the Roche LightCycler 480 software. Gene expression levels were calculated using the 2^−∆∆Ct^ method. The results are shown in Additional file 2: Figures S2 and S3.

## Results

### The chimeric receptor method can provide clues to constitutive homodimerization of LysM-type proteins

Hairy roots of *L. japonicus* expressing a given chimeric receptor pair and a *NIN*p-*GUS* construct (*NIN* promoter fused to the *GUS* reporter gene) were obtain by *A. rhizogenes* mediated transformation. In addition to MG-20 wild-type plants, we also used the Nod factor receptor gene mutants *nfr1-1* and *nfr5-2* (ecotype Gifu) for expression of chimeric receptors in hairy roots. In this system, the function of constitutive and ligand-induced receptor pairs can be analyzed (Fig. 1). Three weeks after *A. rhizogenes* transformation, hairy roots were treated with sterilized water, 1 μM chitoheptaose or 10 μg/mL chitin for 4 h and then stained for GUS activity to monitor *NIN* gene expression. Three independent experiments were performed with the constructed *A. rhizogenes* strains. In total, 377 (experiment 1), 350 (experiment 2) and 351 (experiment 3) hairy roots were formed by 216 MG-20 plants. Furthermore, 402 hairy roots from 90 *nfr1-1* plants and 375 hairy roots from 90 *nfr5-2* plants were analyzed. Details on the number of GUS-positive roots for each test combination are shown in Additional file 1: Tables S3 and S4. The average transformation efficiency of MG-20 plants (as estimated by the ratio of GUS-positive hairy roots to the total number of formed hairy roots) was 48.6 ± 3.6 (Additional file 1: Table S3). Similar values were obtained for the *nfr1-1* (48.3 ± 5.1) and *nfr5-2* (52.6 ± 7.0) mutants (Additional file 1: Table S4). Analysis by reverse transcription PCR and qRT-PCR confirmed expression of the chimeric receptor genes in the hairy roots (Additional file 2: Figures S1, S2 and S3).

**Fig. 2:**
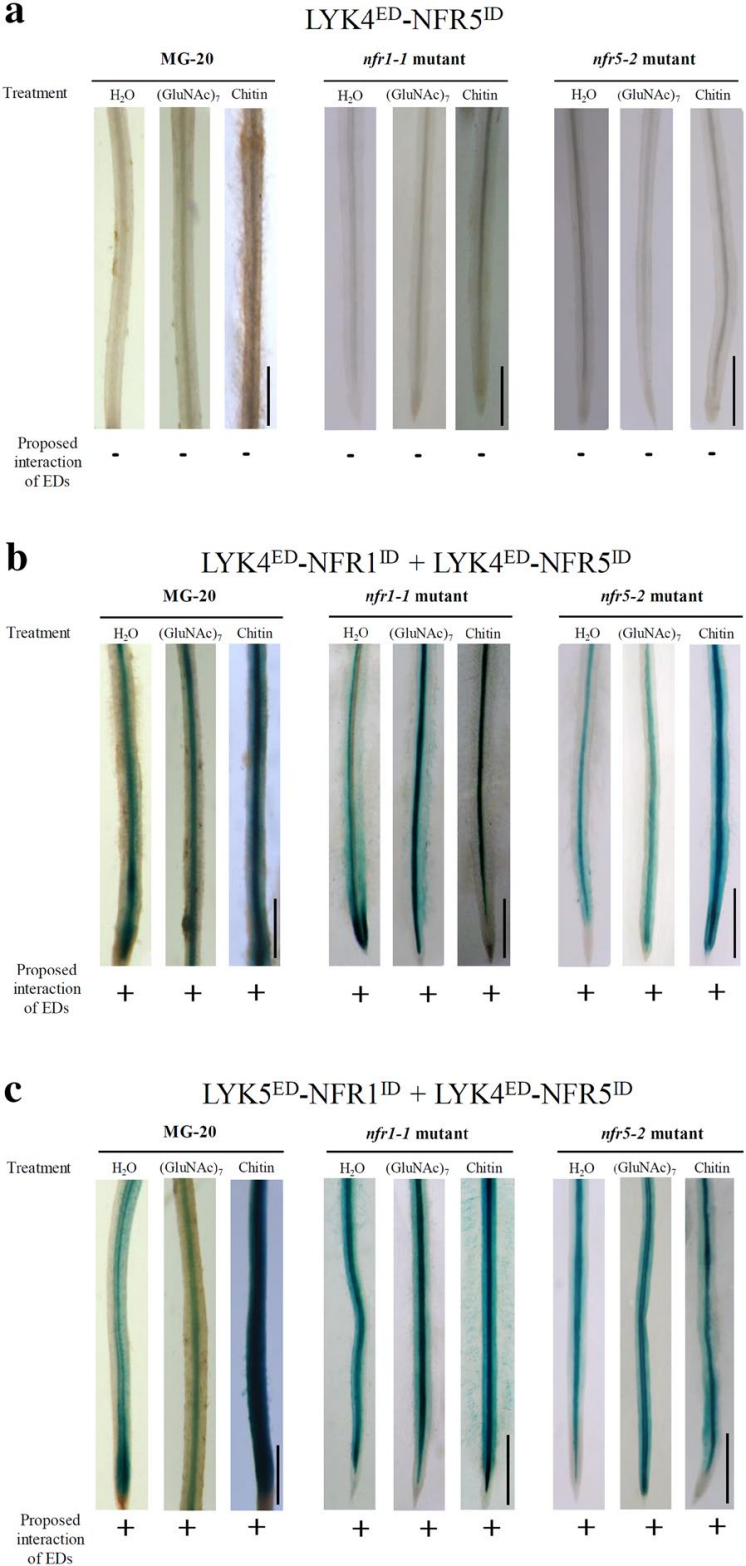
Functional analysis of chimeric receptors in *L. japonicus* can provide information on constitutive dimerization of LysM protein ectodomains (EDs). Indicated chimeric proteins were expressed in roots of ecotype MG-20 and Nod factor receptor gene mutants. A co-transformed *NIN*p-*GUS* construct allowed visualization of receptor-dependent activation of *NIN* expression. Three weeks after transformation, roots were treated with sterile water, 1 μM chitoheptaose [(GluNAc)_7_] or 10 μg/mL chitin for 4 h and finally stained for GUS activity. **a** Roots expressing the single hybrid protein LYK4^ED^–NFR5^ID^ did not show GUS activity. **b** Blue coloration was observed when the proteins LYK4^ED^–NFR1^ID^ and LYK4^ED^–NFR5^ID^ were co-expressed. **c** Analysis of LYK5^ED^–NFR1^ID^ combined with LYK4^ED^–NFR5^ID^ also resulted in blue-colored roots. Bars = 0.8 cm

We first asked whether chimeric receptor analysis can be used to obtain information on constitutive homodimerization, i.e., the ectodomain dimerization of a given LysM-type protein in a ligand-independent manner. The chitin receptor complex protein LYK4 of *A. thaliana* was used as a known example of a LysM-type protein that forms such constitutive homodimers [35]. We therefore examined the ability of LYK4^ED^–NFR1^ID^ and LYK4^ED^–NFR5^ID^ to trigger GUS activity in *L. japonicus* roots. These hybrid receptor proteins consisted of the ectodomain of LYK4 (LYK4^ED^) fused to the intracellular domains of the Nod factor receptors NFR1 (NFR1^ID^) and NFR5 (NFR5^ID^), respectively (Fig. 1b). Roots treated with water, chitoheptaose or chitin were subjected to GUS staining. Control roots transformed with the single hybrid protein LYK4^ED^–NFR5^ID^ (and also carrying the *NIN*p-*GUS* construct) did not show GUS activity (Fig. 2a). In contrast, roots co-expressing LYK4^ED^–NFR1^ID^ and LYK4^ED^–NFR5^ID^ showed blue coloration indicating that *NIN* gene expression was induced (Fig. 2b). Hence, the LYK4^ED^–NFR1^ID^/LYK4^ED^–NFR5^ID^ proteins formed a functional protein complex independently of whether the roots were treated with water or elicitor. These results are consistent with our previous finding that the ectodomain of LYK4 can form constitutive homodimers [35].

### The method also can give information on constitutive heterodimerization of LysM-type proteins

LysM-type proteins can also form constitutive (ligand-independent) heterodimers. We previously found that the ectodomain of LYK4 assembles with the ectodomain of the chitin receptor LYK5 of *A. thaliana* [35]. We therefore asked whether our chimeric receptor approach can provide information on possible constitutive heterodimerization of LysM-type protein ectodomains. We made a DNA construct encoding LYK5^ED^–NFR1^ID^, i.e., the ectodomain of LYK5 was fused to the intracellular domain of NFR1 (Fig. 1b). In contrast to LYK4^ED^–NFR5^ID^ alone (Fig. 2a), co-expression of LYK5^ED^–NFR1^ID^ with LYK4^ED^–NFR5^ID^ in either MG-20, *nfr1-1* or *nfr5-2* plants resulted in blue coloration of GUS-stained roots indicating *NIN* expression. Roots treated with chitoheptaose or chitin showed similar blue coloration (Fig. 2c). Hence, co-expression of the chimeras resulted in a functional receptor pair, confirming the ligand-independent LYK4–LYK5 heterodimerization reported previously [35].

### Analysis of ligand-induced formation of LysM receptor complexes

**Fig. 3:**
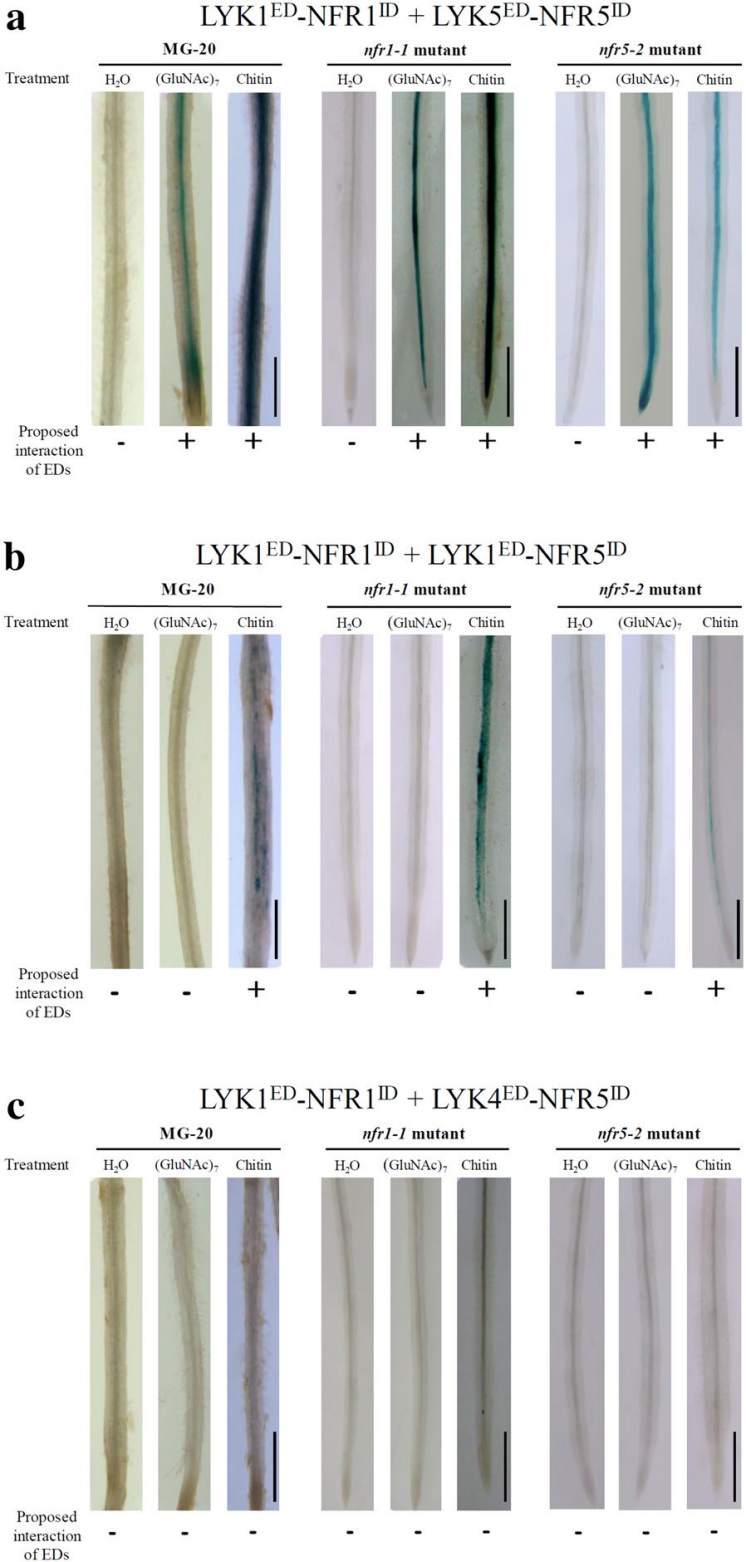
Functional analysis of chimeric receptors in *L. japonicus* can provide information on ligand-induced dimerization of LysM protein ectodomains (EDs). Indicated chimeric proteins were expressed in roots of ecotype MG-20 and Nod factor receptor gene mutants. Co-transformation of the *NIN*p-*GUS* construct provided an estimate for *NIN* gene expression. Prior GUS staining, roots were treated with sterile water, 1 μM chitoheptaose [(GluNAc)_7_] or 10 μg/mL chitin for 4 h. **a** Roots co-expressing LYK1^ED^–NFR1^ID^ with LYK5^ED^–NFR5^ID^ showed blue coloration upon treatment with chitoheptaose or chitin. **b** Co-expression of LYK1^ED^–NFR1^ID^ with LYK1^ED^–NFR5^ID^ resulted in weak blue coloration when roots were treated with chitin. **c** No blue coloration was observed for the combination LYK1^ED^–NFR1^ID^ with LYK4^ED^–NFR5^ID^. Bars = 0.8 cm

Ligand-induced dimerization of LysM receptor proteins is believed to play a primary role in triggering activation of downstream signaling pathways. Previous work showed that chitin signaling in *A. thaliana* is activated by ligand-induced heterodimerization of the chitin receptor complex proteins LYK1 and LYK5 [32, 35]. Here, we investigated whether we could obtain similar results with our chimeric receptor method. *A. rhizogenes* carrying plasmids encoding LYK1^ED^–NFR1^ID^ (ectodomain of LYK1 fused to the intracellular domain of NFR1) and LYK5^ED^–NFR5^ID^ (ectodomain of LYK5 fused to the intracellular domain of NFR5) (Fig. 1b) were used for root transformation of the different *L. japonicus* genotypes. The formed roots were exposed to water, chitoheptaose or chitin and finally stained for GUS activity reflecting *NIN* expression. As shown in Fig. 3a, the water treatment did not result in blue coloration of the roots whereas application of either chitoheptaose or chitin caused strong blue coloration. These results indicate a ligand-induced interaction between LYK1^ED^ and LYK5^ED^ and thus confirm previous findings on the LYK1–LYK5 protein complex formation [32, 35].

Previous work suggested that chitin-induced homodimerization of LYK1 also can activate chitin signaling in *A. thaliana* [27]. We therefore asked whether a chimeric receptor pair with ectodomains of LYK1 alone is functional in the chimeric receptor system. The combination LYK1^ED^–NFR1^ID^ with LYK1^ED^–NFR5^ID^ (ectodomain of LYK1 fused to the intracellular domain of NFR1 and NFR5, respectively; Fig. 1b) was investigated. Transformed roots treated with water or chitoheptaose showed no visible blue coloration, indicating that *NIN* expression was not activated. However, blue coloration, often relatively faint, was observed when roots were treated with chitin (Fig. 3b). These findings suggest that chitin-induced LYK1 homodimerization was possible as reported previously [27].

### Analysis of non-functional receptor pairs

In contrast to LYK5, LYK4 did not directly interact with LYK1 in our previous immunoprecipitation and BiFC experiments [35]. The chimeric receptor method provided similar results. The transformed *L. japonicus* roots co-expressing LYK1^ED^–NFR1^ID^ and LYK4^ED^–NFR5^ID^ were analyzed as stated above. As expected, the examined receptor combination was non-functional. Roots co-expressing LYK1^ED^–NFR1^ID^ and LYK4^ED^–NFR5^ID^ showed no visible GUS staining reflecting *NIN* expression. Likewise, roots treated with chitoheptaose or chitin failed to induce GUS activity (Fig. 3c). Hence, the chimeric receptor method also could provide information on the lack of direct LysM-type protein interactions.

## Discussion

In this article, we describe a method that tests functionality of chimeric LysM receptor protein pairs expressed in *L. japonicus* roots. This method was originally developed to analyze the chitin-induced interaction between the ectodomains of the receptor proteins OsCERK1 and OsCEBiP in rice [33]. Here we show that the method also can provide information on ligand-independent homodimerization and heterodimerization of LysM receptor proteins. Hence, the method allows discrimination between constitutive and ligand-induced protein–protein interactions. Furthermore, negative results can be obtained, indicating that the examined ectodomains could not form a functional receptor complex.

The described method is based on expression of chimeric receptors that activate Nod factor signaling in *L. japonicus* [40]. Nod factor signaling in legumes results in up-regulation of symbiosis-related genes such as *NIN*, a key transcriptional regulator required for expression of other symbiotic genes [17]. Here we used a *NIN*p*-GUS* construct to estimate *NIN* expression in *L. japonicus*. In addition, transcript levels of other symbiotic genes, such as the GRAS transcriptional regulator genes *NSP1* and *NSP2*, can be determined by qRT-PCR as reported previously [33]. We recommend analyzing *NIN* expression as a prime response marker because constitutive GUS activity reflecting *NIN* expression was not detected in any control plants in our current and previous [33] experiments.

The chimeric constructs were expressed from the CaMV 35S promoter to allow sufficient expression of the protein pairs in equal amounts. This promoter has been successfully used to analyze ligand-induced heterodimerization of rice chitin receptor proteins in our previous study [33]. The CaMV 35S promoter is often used to study protein–protein interactions *in planta*, including ligand-induced heterodimerization of LYK1 with LYK5 in *A. thaliana* [32, 35]. Furthermore, it is recommended analyzing additional *L. japonicus* control roots that express a single hybrid protein as performed with LYK4^ED^–NFR5^ID^ in this study and with rice chitin receptor constructs in our previous work [33]. In all examined cases, the single constructs did not cause GUS activation, indicating that the roots showed no endogenous (receptor-independent) *NIN* expression. Hence, the described method is robust and suitable to analyze functionality of specific LysM-type receptor pairs.

The described method requires co-expression of two chimeric receptor genes and a *NIN*p*-GUS* construct in *L. japonicus*. GUS staining reflecting *NIN* promoter activity can be considered as a measure for successful co-transformation of the expressed constructs. It is also recommended to confirm expression of the chimeric receptor genes by qRT-PCR. The transformation efficiency (as estimated by the ratio of GUS-positive hairy roots to the total number of formed hairy roots) was nearly 50% under the described test conditions. Co-expression of fluorescence proteins and removing of non-fluorescent hairy roots may further increase the transformation efficiency. However, we do not recommend the use of selection media containing antibiotics or herbicides, because Nod factor signaling in *L. japonicus* may be impaired under such conditions.

In our previous study on chitin receptors from rice, *L. japonicus* Gifu lines were used that have been stably transformed with a *NIN*p*-GUS* construct [33]. However, ecotype Gifu plants were difficult to propagate under our growth room and greenhouse conditions. We therefore simplified the system by cloning the chimeric receptor gene constructs into a binary vector containing *NIN*p*-GUS* (pCAMBIA-*NIN*p). Thus, any *L. japonicus* genotype with a functional Nod factor signaling pathway, including the early-flowering ecotype MG-20 suitable for indoor handling [41], can now be used.

The current study shows that the GUS staining results with MG-20 wild-type plants and the Gifu Nod factor receptor gene mutants (*nfr1-1* and *nfr5-2*) were similar, indicating that the presence of the full-length NFR1 and NFR5 proteins in MG-20 did not influence the interactions of the examined LYK ectodomains of *A. thaliana*. These findings indicate that induction of non-specific (NFR1 and NFR5 mediated) downstream signaling was absent or below the detection limit. This is not surprising because NFR1 and NFR5 have been reported to specifically perceive Nod factors to induce downstream signaling [15, 16]. Hence, we suggest that MG-20 plants can be used for testing any LysM-type receptor pair with the described method provided the ligand is not a lipo-chitooligosaccharide. In case of characterization of Nod factor receptors and the use of Nod factors, however, the chimeric receptor analysis should be performed with Nod factor receptor gene mutants of *L. japonicus*.

Whole roots were treated with chitoheptaose and chitin in our experiments. Alternatively, roots can be spot-treated with a given elicitor as reported previously [33]. For elicitor treatment of hairy *L. japonicus* roots, we recommend using an incubation time of 4 h. We have chosen this value because a previous time course experiment with chimeric receptors containing ectodomains of OsCERK1 and OsCEBiP showed increased *NIN* promoter activity as early as 3 h when roots were treated with chitooctaose. Blue coloration was first observed in the rhizodermis and later in cortical and vascular cells of the roots [33]. In the present study, blue coloration was seen in epidermal, cortical and vascular cells to different extents and was often strongest in the vascular tissue. Differences in GUS activity could be due to insertion of the *NIN*p*-GUS* construct into different genomic regions. Moreover, different levels of phytohormones in the hairy roots could eventually have modulated the strength of GUS activity, particularly in the central vascular tissue. We therefore recommend analyzing hairy roots from several plants. Furthermore, clearing of GUS-stained roots with commercial bleach made the blue coloration within the root more visible but eventually had an attenuating effect on GUS staining at the root surface. Observed tissue-specific variations and quantitative differences in GUS staining intensities should therefore not be over-interpreted.

Immunoprecipitation and BiFC analysis in previous studies revealed constitutive and ligand-induced interactions between LYK proteins of *A. thaliana* [27, 32, 35]. The results of the described chimeric receptor method were entirely consistent with these previous findings and provide additional evidence for constitutive homodimerization of LYK4^ED^–LYK4^ED^ [35], constitutive heterodimerization of LYK4^ED^–LYK5^ED^ [35] and ligand-induced heterodimerization of LYK1^ED^–LYK5^ED^ [32, 35]. Taken these findings together, we conclude that chitin signaling in *A. thaliana* is triggered by formation of a tripartite chitin receptor complex consisting of LYK1, LYK4 and LYK5. Upon ligand binding, LYK1^ED^ interacts with LYK5^ED^ from the LYK4^ED^–LYK5^ED^ heterodimer as illustrated in Additional file 2: Figure S4. Furthermore, our results support the possibility that a homodimeric LYK1^ED^–LYK1^ED^ complex can be formed in response to chitin treatment [27]. However, chitin-induced LYK1^ED^–LYK1^ED^ dimerization is perhaps rather weak and chitoheptaose was obviously too short for receptor dimerization. Moreover, in agreement with our previous analysis [35], no indications for a functional LYK1^ED^–LYK4^ED^ interaction were found with the chimeric receptor method.

## Conclusion

The chimeric receptor approach described in this article provides information on interactions between ectodomains of LysM-type receptor proteins. Taking LYK1, LYK4, LYK5 as examples, we used the method to verify constitutive and ligand-induced interactions between chitin receptor proteins of *A. thaliana*. The method can be applied to obtain clues for homodimerization of a given LysM-type protein or to test specific candidate receptor pairs for functionality. In future, the method could serve as a powerful tool to identify and characterize co-receptors or scaffold proteins in receptor complexes. In case of ligand-induced dimerization, the method also could be used to identify novel microbial signals perceived by a chimeric receptor pair of hitherto unknown function. Future work will be required to examine the possibility to modify the described method for testing receptor protein–protein interactions in general.

## Supplementary information

**Additional file 1: Table S1.** List of primers used in this study. **Table S2.** List of plasmids used in this study. **Table S3.** Transformation efficiency of *L. japonicus* MG-20 as estimated by the ratio of GUS-positive hairy roots to the total number of formed hairy roots. **Table S4.** Transformation efficiency of the *L. japonicus* mutants *nfr1-1* and *nfr5-2* as estimated by the ratio of GUS-positive hairy roots to the total number of formed hairy roots.

**Additional file 2: Figure S1.** RT-PCR analysis of chimeric receptor genes expressed in* L. japonicus* roots. **Figure S2.** qRT-PCR analysis of chimeric receptor gene pairs in *L. japonicus* roots showing activation of* NIN* expression. **Figure S3.** qRT-PCR analysis of chimeric receptor gene constructs in *L. japonicus* roots lacking activation of *NIN* expression. **Figure S4.** Model for chitin receptor protein interactions in *A. thaliana*.

## Data Availability

Vectors and MG-20 seeds are available from the corresponding authors. *L. japonicus* Nod factor receptor gene mutants should be requested from Simona Radutoiu or Jens Stougaard (Department of Molecular Biology and Genetics, Aarhus University, DK-8000 Aarhus, Denmark).
