# Supplementary material for: A method for functional testing constitutive and ligand-induced interactions of lysin motif receptor proteins
Source: Plant Methods. 2020 Jan 16;16:3. doi: 10.1186/s13007-020-0551-4 (PMC6964010; doi:10.1186/s13007-020-0551-4)
Supplement: Supplementary file 2 — Additional file 2: Figure S1. RT-PCR analysis of chimeric receptor genes expressed in L. japonicus roots. Figure S2. qRT-PCR analysis of chimeric receptor gene pairs in L. japonicus roots showing activation of NIN expression. Figure S3. qRT-PCR analysis of chimeric receptor gene constructs in L. japonicus roots lacking activation of NIN expression. Figure S4. Model for chitin receptor protein interactions in A. thaliana. [file 13007_2020_551_MOESM2_ESM.pdf]

Additional file 2

A method for functional testing constitutive and ligand-induced interactions of lysin motif receptor proteins

Chun-Lian Li, De-Xing Xue, Yi-Han Wang, Zhi-Ping Xie and Christian Staehelin

This file contains additional figures:
Figure S1 RT-PCR analysis of chimeric receptor genes expressed in *L. japonicus* roots.
Figure S2 qRT-PCR analysis of chimeric receptor gene pairs in *L. japonicus* roots showing activation of *NIN* expression.
Figure S3 qRT-PCR analysis of chimeric receptor gene constructs in *L. japonicus* roots lacking activation of *NIN* expression.
Figure S4 Model for chitin receptor protein interactions in *A. thaliana*.

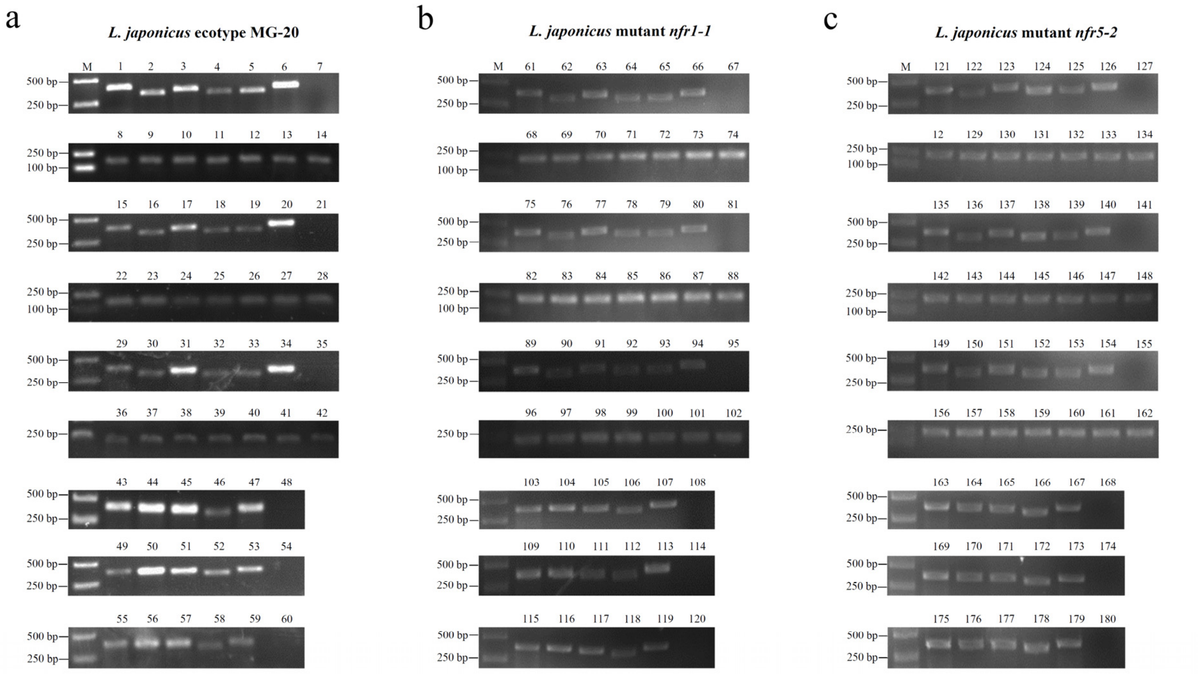

Fig. S1 RT-PCR analysis of chimeric receptor genes expressed in *L. japonicus* roots. *A. rhizogenes*-mediated transformation was used to express chimeric receptor genes in roots of *L. japonicus* ecotype MG-20 (**a**), the Gifu mutant *nfr1-1* (**b**) and the Gifu mutant *nfr5-2* (**c**). Four hairy roots (from 4 plants) were combined to obtain an RNA sample. RNA samples were extracted from transformed roots that were treated with 10 µg/mL chitin (lanes 1-14, 43-48; 61-74, 103-108; 121-134 and 163-168), 1 µM $(GluNAc)_7$ (chitoheptaose; hepta-*N*-acetylchitoheptaose) (lanes 15-28, 49-54; 75-88, 109-114; 135-148 and 169-174) or sterile water (lanes 29-42, 55-60; 89-102, 115-120; 149-162 and 175-180). Roots were subjected to GUS staining 4 h after the treatment. Primers used to detect expression of the chimeric receptor genes are listed in Additional file 1 (Table S1; Primers 23 to 31). Different forward primers were used when co-expression of chimeric receptor genes with the same ectodomain was analyzed. In roots expressing $LYK4^{ED}$-$NFR5^{ID}$ (as in Fig. 2a), $LYK4^{ED}$-$NFR5^{ID}$ expression was analyzed with primers 27 and 24 (375 bp amplicon; lanes 3, 17, 31, 63, 77, 91, 123, 137 and 151). In roots expressing $LYK4^{ED}$-$NFR1^{ID}$ and $LYK4^{ED}$-$NFR5^{ID}$ (as in Fig. 2b), $LYK4^{ED}$-$NFR1^{ID}$ expression was analyzed with primers 28 and 23 (320 bp amplicon; lanes 5, 19, 33, 65, 79, 93, 125, 139 and 153), and $LYK4^{ED}$-$NFR5^{ID}$ expression was analyzed with primers 28 and 24 (321 bp amplicon; lanes 46, 52, 58, 106, 112, 118, 166, 172 and 178). In roots expressing $LYK5^{ED}$-$NFR1^{ID}$ and $LYK4^{ED}$-$NFR5^{ID}$ (as in Fig. 2c), $LYK5^{ED}$-$NFR1^{ID}$ expression was analyzed with primers 29 and 23 (387 bp amplicon; lanes 6, 20, 34, 66, 80, 94, 126, 140 and 154), and $LYK4^{ED}$-$NFR5^{ID}$ expression was analyzed with primers 27 and 24 (375 bp amplicon; lanes 47, 53, 59, 107, 113, 119, 167, 173 and 179). In roots

expressing *LYK1*^{ED}-*NFR1*^{ID} and *LYK5*^{ED}-*NFR5*^{ID} (as in Fig. 3a), *LYK1*^{ED}-*NFR1*^{ID} expression was analyzed with primers 25 and 23 (347 bp amplicon; lanes 2, 16, 30, 62, 76, 90, 122, 136 and 150), and *LYK5*^{ED}-*NFR5*^{ID} expression was analyzed with primers 29 and 24 (383 bp amplicon; lanes 44, 50, 56, 104, 110, 116, 164, 170 and 176). In roots expressing *LYK1*^{ED}-*NFR1*^{ID} and *LYK1*^{ED}-*NFR5*^{ID} (as in Fig. 3b), *LYK1*^{ED}-*NFR1*^{ID} expression was analyzed with primers 26 and 23 (391 bp amplicon; lanes 1, 15, 29, 61, 75, 89, 121, 135 and 149), and *LYK1*^{ED}-*NFR5*^{ID} expression was analyzed with primers 26 and 24 (387 bp amplicon; lanes 43, 49, 55, 103, 109, 115, 163, 169 and 175). In roots expressing *LYK1*^{ED}-*NFR1*^{ID} and *LYK4*^{ED}-*NFR5*^{ID} (as in Fig. 3c), *LYK1*^{ED}-*NFR1*^{ID} expression was analyzed with primers 25 and 23 (347 bp amplicon; lanes 4, 18, 32, 64, 78, 92, 124, 138 and 152), and *LYK4*^{ED}-*NFR5*^{ID} expression was analyzed with primers 27 and 24 (375 bp amplicon; lanes 45, 51, 57, 105, 111, 117, 165, 171 and 177). The *Ubiquitin* gene (Lj5g3v2060710.1) primers 30 and 31 were used for control reactions (230 bp amplicon; lanes 8-14, 22-28, 36-42; 68-74, 82-88, 96-102; 128-134, 142-148, and 156-162). Non-transformed roots were used as a negative control (lanes 7, 14, 21, 28, 35, 42, 48, 54, 60, 67, 74, 81, 88, 95, 102, 108, 114, 120; 127, 134, 141, 148, 155, 162, 168, 174 and 180).

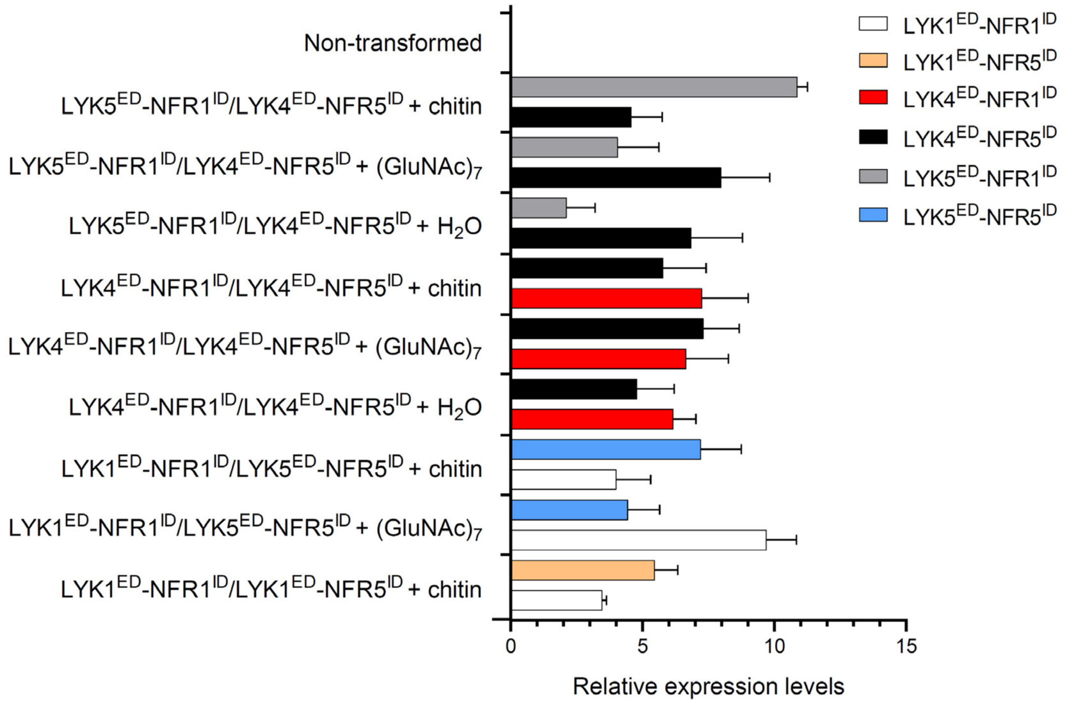

Fig. S2 qRT-PCR analysis of chimeric receptor gene pairs in *L. japonicus* roots showing activation of *NIN* expression. Transformed hairy roots (ecotype MG-20) were treated with chitin, chitoheptaose or water. RNA was isolated from transgenic roots showing blue coloration after GUS staining. Four to five hairy roots (from different plants) were combined to obtain an RNA sample. Primers used for expression analysis are listed in Additional file 1 (Table S1). *LYK1^{ED}-NFR1^{ID}* expression was analyzed with primers 32 and 35, *LYK1^{ED}-NFR5^{ID}* with primers 32 and 36, *LYK4^{ED}-NFR1^{ID}* with primers 33 and 35, *LYK4^{ED}-NFR5^{ID}* with primers 33 and 36, *LYK5^{ED}-NFR1^{ID}* with primers 34 and 35, and *LYK5^{ED}-NFR5^{ID}* with primers 34 and 36. Non-transformed roots served as a negative control (no detection of transcripts). Data indicate means ± SE from three independent RNA isolations (n=3).

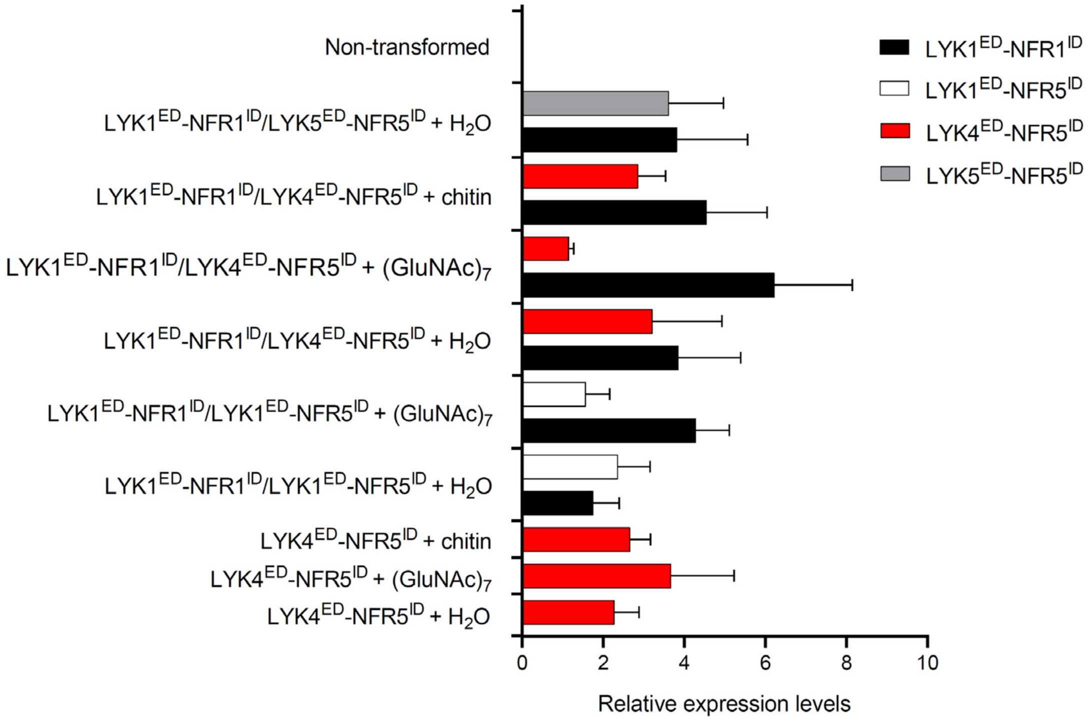

Fig. S3 qRT-PCR analysis of chimeric receptor gene constructs in *L. japonicus* roots lacking activation of *NIN* expression. Transformed roots (ecotype MG-20) were treated with chitin, chitoheptaose or water. Four to five hairy roots (from different plants) were combined to obtain an RNA sample. Primers used for expression analysis are listed in Additional file 1 (Table S1). *LYK1*^{ED}-*NFR1*^{ID} expression was analyzed with primers 32 and 35, *LYK1*^{ED}-*NFR5*^{ID} with primers 32 and 36, *LYK4*^{ED}-*NFR1*^{ID} with primers 33 and 35, *LYK4*^{ED}-*NFR5*^{ID} with primers 33 and 36, *LYK5*^{ED}-*NFR1*^{ID} with primers 34 and 35, and *LYK5*^{ED}-*NFR5*^{ID} with primers 34 and 36. Non-transformed roots served as a negative control (no detection of transcripts). Data indicate means ± SE from three independent RNA isolations (n=3).

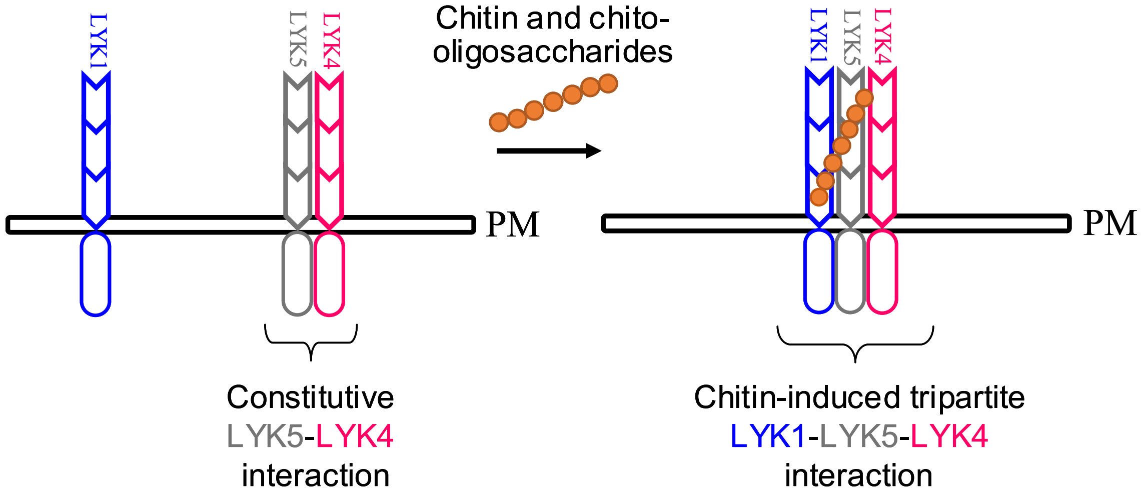

Fig. S4 Model for chitin receptor protein interactions in *A. thaliana*. LYK4 can form constitutive heterodimers with LYK5. Upon binding to chitin or chitooligosaccharides, LYK1 interacts with LYK5. Formation of a tripartite receptor complex triggers downstream signaling. PM, plasma membrane.
